# Transarterial Chemoembolization Combined With Apatinib for Advanced Hepatocellular Carcinoma: A Propensity Score Matching Analysis

**DOI:** 10.3389/fonc.2020.00970

**Published:** 2020-07-07

**Authors:** Xuefeng Kan, Bin Liang, Guofeng Zhou, Bin Xiong, Feng Pan, Yanqiao Ren, Yanyan Cao, Jihua Wang, Fan Yang, Chuansheng Zheng

**Affiliations:** ^1^Department of Radiology, Union Hospital, Tongji Medical College, Huazhong University of Science and Technology, Wuhan, China; ^2^Hubei Provinve Key Laboratory of Molecular Imaging, Wuhan, China

**Keywords:** apatinib, transarterial chemoembolization, hepatocellular carcinoma, propensity score matching, adverse effects

## Abstract

**Background:** Apatinib is a powerful inhibitor of vascular endothelial growth factor receptor-2. This study was aimed to investigate whether apatinib could improve the efficacy of transarterial chemoembolization (TACE) in patients with advanced hepatocellular carcinoma (HCC).

**Methods:** Between June 2015 and September 2018, 357 patients with HCC at Barcelona Clinic Liver Cancer stage C who received the treatment of TACE combining with apatinib (TACE–apatinib) or TACE-alone were included. Propensity score matching (PSM) analysis was used to reduce the patient selection bias.

**Results:** Ninety pairs of patients were chosen after the PSM analysis. The disease control rates of tumor and a-fetoprotein response in the TACE–apatinib group were significantly higher than that of the TACE-alone group before and after the PSM analysis (*P* < 0.05). Before the PSM analysis, the median time of tumor progression (TTP) and the overall survival (OS) in the TACE–apatinib group were significantly greater than those of the TACE-alone group (TTP: 9.0 months vs. 3.0 months, *P* < 0.001; OS: 14.0 months vs. 7.0 months, *P* < 0.001). After the PSM analysis, the median TTP and OS in the TACE–apatinib group was also significantly greater than that of the TACE-alone group (TTP: 7.0 months vs. 3.0 months, *P* < 0.001; OS: 13.0 months vs. 8.0 months, *P* < 0.001); the uni- and multivariate analysis revealed that TACE–apatinib was a protective factor for OS. Fourteen patients emerged with grade 3 apatinib-related adverse events.

**Conclusion:** The efficacy of TACE–apatinib for patients with advanced HCC was inspiring, and the side effects of apatinib were tolerable.

## Introduction

Hepatocellular carcinoma (HCC) is one of the most common cancers with one of the highest mortality in the world and a high morbidity in China (1, 2). Unfortunately, about 40% of patients with HCC still only depend on palliative treatment for diagnosing at an advanced stage [Barcelona Clinic Liver Cancer (BCLC) stage C] (3, 4). Sorafenib, a multi-kinase inhibitor and the first molecular targeted drug for HCC, was recommended as the first-line treatment for advanced HCC (1). However, its efficacy for Asian patients with advanced HCC was not very satisfactory, with the median overall survival (OS) at 6.5 months and the objective response rate at 3.3% (5).

Transarterial chemoembolization (TACE), a common and effective palliative treatment for patients with unresectable HCC, was recommended as the standard treatment for patients with BCLC stage B HCC (1). However, for some selected patients with BCLC stage C HCC, some studies (6–8) suggested that these patients could get survival benefits from TACE treatment, and TACE was also safe for these patients. Furthermore, an increased interest in a treatment combining TACE with sorafenib, to improve the efficacy in patients with advanced HCC, was shown in some previous studies (9–11).

Apatinib is a powerful inhibitor of vascular endothelial growth factor receptor-2 (VEGFR-2) (12). It was approved in the treatment of patients with advanced gastric cancer in China (13). Meanwhile, several previous studies (14–16) indicated that apatinib showed inspiring anti-tumor activities in patients with ovarian cancer, breast cancer, and HCC, respectively. Therefore, we assumed that apatinib may improve the efficacy of TACE by inhibiting the revascularization of tumor in the treatment of advanced HCC. In this retrospective control study, a propensity score matching (PSM) analysis was conducted to investigate whether apatinib could improve the efficacy of TACE in patients with advanced HCC (BCLC stage C).

## Materials and Methods

### Study Design and Patient Selection

The study continued from June 2015 to September 2018, and it included 357 consecutive patients with BCLC stage C HCC who received the treatment of TACE combined with apatinib (TACE–apatinib) or TACE alone. The method of PSM analysis was used to reduce the patient selection bias and balance the variables between the two different treatment groups. The safety and the efficacy of the two treatment methods in patients with advanced HCC were retrospectively investigated before and after the PSM analysis. Approval for this retrospective study was obtained from the Ethics Committee of Tongji Medical College, Huazhong University of Science and Technology. A written informed consent was also obtained from each patient before the first TACE procedure.

The patients who were included in this study were (1) patients who were diagnosed with HCC according to a pathologic examination or noninvasive criteria in accordance with the American Association for the study of Liver Disease guidelines (17)/European Association for the Study of Liver, (2) patients with HCC in BCLC stage C, (3) patients with liver function graded at Child–Pugh A or B, (4) patients with Eastern Cooperative Oncology Group (ECOG) score of ≤2 points (18), (5) patients with platelet count ≥60 × 10^9^ platelets per liter, neutrophil count >1.5 × 10^9^ cells per liter, and hemoglobin >9 g/dl (19), and (6) patients with aspartate aminotransferase and alanine aminotransferase <200 U/L and total bilirubin ≤50 umol/l (19).

The patients who were excluded from the study were (1) patients with complete occlusion of the main portal vein (18), (2) patients with moderate or severe ascites, (3) patients who had previously undergone a treatment of oral sorafenib, liver resection, systemic chemotherapy, transarterial chemoinfusion, or TACE, (4) patients with serious comorbidities, such as severe dysfunction of the kidney, lung, or heart, and (5) patients who received other therapies during this study, such as iodine 125 seed implantation, radiofrequency ablation, external beam radiotherapy, or percutaneous ethanol injection.

### TACE Procedure

The operators (XK, GZ, BX, BL, FP, and CZ) of the TACE procedures had at least 8 years of experience in performing TACE procedures. Initially, the tip of a 3-French microcatheter (Progreat, Terumo, Tokyo, Japan) or a 5-French catheter (Cook, Bloomington, IN, USA) was introduced into the tumor-feeding arteries. Then, 10–20 ml of lipiodol (Lipiodol Ultrafluido, Guerbet, France) was mixed with 20–40 mg of doxorubicin hydrochloride (Hisun Pharmaceutical Co. Ltd., Zhejiang, China) to create an emulsion. Based on the tumor size and the liver function, 5–20 ml of the emulsion was injected into the tumor-feeding arteries through a 5-French catheter or a 3-French microcatheter. Lastly, gelatin sponge particles (300–700 um, Cook, USA) were used to supplement embolization until the stagnation of artery flow appeared. For patients with arterioportal shunt, polyvinyl alcohol particles (300–1,000 um, Cook, USA) were used for blocking the shunt before infusion of the emulsion of lipiodol and doxorubicin.

### Apatinib Administration

In the TACE–apatinib group, apatinib was orally taken 3–5 days after each TACE procedure. The initial dose for each patient was 500 mg/day. The dose reduction of apatinib was based on the patients' tolerance to the drug. The grade of the adverse events of apatinib was defined based on the National Cancer Institute Common Terminology Criteria for Adverse Events (version 4.0). If the adverse events of apatinib were equal to or greater than grade 3, the dose of apatinib was reduced to 250 mg/day to relieve or eliminate the adverse events. If the adverse events (≥ grade 3) continued after the dose adjustment or apatinib-related adverse events of gastrointestinal hemorrhage occurred, the administration of the drug was temporarily interrupted. When the adverse events were relieved or eliminated, the dose was changed to 250 mg/day.

### Follow-Up and Repeated TACE

All the patients received a series of follow-up until March 31, 2019. A physical examination, either an abdominal contrast-enhanced computed tomography (CT) or contrast-enhanced magnetic resonance (MR), and a digital subtraction angiography imaging of the hepatic artery were included in the series of follow-up. Laboratory tests included urine and hematologic analyses. Data on proteinuria, prothrombin time, a-fetoprotein (AFP), total bilirubin, serum albumin, thyroid-stimulating hormone, triiodothyronine (T3), thyroxine (T4), and free T4 were collected at each follow-up. The first follow-up was conducted at 4 weeks after the first TACE procedure. If an intrahepatic recurrent tumor or residual viable tumor was revealed by the contrast-enhanced CT or MR images, a repeated TACE was performed if the patients had no contraindication of TACE. The patients received continuous apatinib with no breaks before the repeated TACE and an interruption of apatinib for 3–5 days after the repeated TACE. The next follow-up interval was then extended to every 2 months after the initial follow-up at 4 weeks after the first TACE procedure.

### Assessments

The adverse events of apatinib were assessed based on the National Cancer Institute Common Terminology Criteria for Adverse Events (version 4.0). The value of albumin, the total serum bilirubin level, and the prothrombin time, which were obtained at 4 weeks after the first TACE procedure in the TACE–apatinib group, were used to investigate the impact of treatment on liver function. In the two treatment groups, the adverse events that related to TACE were recorded from the second TACE procedure. Tumor response was evaluated based on the modified Response Evaluation Criteria in solid tumors (20), and it was assessed by an experienced radiologist (FY, with more than 15 years of experience). Meanwhile, the survival data and the treatment information were concealed from the radiologist. AFP response at 4 weeks after the first TACE procedure in the two groups was assessed as follows: (1) complete response (decrease to normal), (2) partial response (decrease by >50% of the baseline value), (3) stable (change between−50% and +50% of the baseline value), or (4) progression (increase by >50% of the baseline value) (21). The disease control rates (DCR) of the tumor and the AFP response were defined as the percentage of patients with a response rate of complete response, partial response, and stable.

In addition, the time of tumor progression (TTP) and the OS of patients in the two groups were recorded, respectively. TTP was defined as the time from the start of the first TACE procedure to the time of tumor progression. OS was defined as the time from the first TACE procedure to the last follow-up or a patient's death.

### Classification of Portal Vein Tumor Thrombus

Portal vein tumor thrombus (PVTT) was defined as that HCC which invaded the portal vein. The types of PVTT were classified into three subgroups: (1) type A, which was defined as PVTT in the main portal vein, (2) type B, which was defined as PVTT in the first-order portal vein branch (to the left or the right portal vein), and (3) type C, which was defined as PVTT in the second or lower-order portal vein branch.

### PSM Analysis

A PSM analysis was conducted in this study to reduce the patient selection bias and balance the variables between the two groups. The baseline variables including gender, age, ECOG performance, HBV infection, Child–Pugh class, mild ascites, AFP level, total values of bilirubin, albumin, and PVTT, hepatic vein tumor thrombus (defined as HCC that invaded the hepatic vein.), and extrahepatic spread were matched in our model. One-to-one matching without replacement was applied, and the value of the caliper was 0.05 (22).

### Statistical Analysis

All the analyses were performed using SPSS version 24.0 software (IBM, Armonk, NY, USA). Continuous variables were summarized as mean ± standard deviation. Pearson *x*^2^ test, correction *x*^2^ test, Fisher's exact test, independent-samples *t* test, and Mann–Whitney U test were all used for a comparison of the baseline characteristics between the two groups. The correction *x*^2^ test and Fisher's exact test were used to compare the occurrence rates of adverse events which are related to TACE between the two groups. The Wilcoxon signed-rank test was used to determine the difference in values of the total bilirubin, serum albumin level, and prothrombin time before and after treatment in the TACE–apatinib group. The OS and TTP curves were obtained using the Kaplan–Meier method, and the differences between the two groups were compared by a log-rank test. Multivariate analysis was performed using the Cox regression model for variables that were found to be significant in the univariate analysis, and the risk factors that affected the OS were determined. All statistical tests were two-sided, with a statistically significant value of *P* < 0.05.

## Results

### Study Population

**Figure 1:**
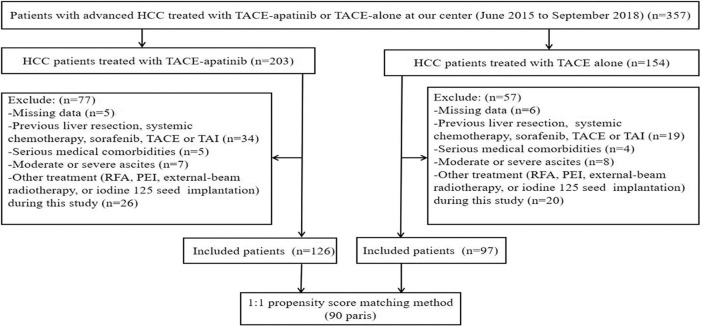
Flow diagram of patient selection. HCC, hepatocellular carcinoma; TACE, transarterial chemoembolization; TACE–apatinib, TACE combined with apatinib; TAI, transarterial chemoinfusion; RFA, radiofrequency ablation; PEI: percutaneous ethanol injection.

A total of 357 patients with BCLC stage C HCC underwent a treatment of either TACE–apatinib or TACE alone in this study. However, 134 patients were excluded from the study. Thus, 223 patients were included in this analysis: 126 patients underwent the treatment of TACE–apatinib, and 97 patients underwent the TACE-alone treatment. After the PSM analysis, 90 pairs of patients were matched (Figure 1). The baseline characteristics of patients who were included in this study before and after the PSM analysis are shown in Table 1. The baseline characteristic of age was significantly different between the two groups before the PSM analysis. All the baseline characteristics between the two groups were balanced after the PSM analysis.

**Table 1:** The baseline characteristics between the two groups before and after PSM analysis.

**Characteristics**	**Before PSM**		***P* value**	**After PSM**		***P* value**
	**TACE–apatinib(*n* = 126)**	**TACE alone (*n* = 97)**		**TACE–apatinib (*n* = 90)**	**TACE alone (*n* = 90)**	
Age (years)	50.5 ± 10.3	53.7 ± 10.3	0.025	52.7 ± 9.7	53.1 ± 10.1	0.782
Gender			0.243			0.829
Male	112	81		77	78	
Female	14	16		13	12	
ECOG performance			0.770			0.844
1	102	77		74	75	
2	24	20		16	15	
HBV infection			0.257			0.816
Yes	114	83		80	79	
No	12	14		10	11	
Child–Pugh class			0.850			0.661
A	108	84		79	77	
B	18	13		11	13	
Mild ascites			0.807			0.808
Absent	113	86		80	81	
Present	13	11		10	9	
AFP (ng/ml)			0.792			0.456
>400	62	46		47	42	
≤400	64	51		43	48	
Total bilirubin (μmol/L)	19.0 ± 7.8	18.7 ± 9.7	0.178	19.4 ± 9.4	18.8 ± 9.8	0.404
Albumin (g/L)	36.5 ± 5.1	36.9 ± 5.1	0.619	37.1 ± 5.1	37.0 ± 5.3	0.875
PVTT			0.480			0.925
Absent	56	35		37	33	
Type A	10	7		6	6	
Type B	35	36		31	32	
Type C	25	19		16	19	
HVTT			0.960			>0.99
Absent	118	91		86	85	
Present	8	6		4	5	
Extrahepatic spread						
Absent	64	47	0.729	44	45	0.881
Lymph nodes	32	25	0.949	21	22	0.861
Lung	24	21	0.631	19	19	>0.99
Bones	2	3	0.767	2	3	>0.99
Suprarenal gland	6	2	0.477	6	2	0.278

*Unless indicated, data are numbers of patients*.

*PSM, propensity score matching; ECOG, Eastern Cooperative Oncology Group; HBV, hepatitis B virus; AFP, a-fetoprotein; TACE, transarterial chemoembolization; PVTT, Portal vein tumor thrombus; HVTT, Hepatic vein tumor thrombus*.

### Safety Comparison Between the Groups of TACE–Apatinib and TACE Alone

The adverse events which were related to TACE from the second TACE procedure in the two groups are shown in Table 2. Before the PSM analysis, six adverse events occurred across six patients in the TACE–apatinib group, while eight adverse events occurred across eight patients in the TACE-alone group. After the PSM analysis, four adverse events occurred across four patients in the TACE–apatinib group, and eight adverse events occurred across eight patients in the TACE-alone group. The occurrence rates of these adverse events between the two groups did not exhibit significant differences before and after the PSM analysis. In addition, the liver function changes at 4 weeks after the treatment in the TACE–apatinib group were assessed. The three representative indicators of liver function at 4 weeks after the TACE–apatinib treatment were not significantly different from the baseline values before and after the PSM analysis (Table 3).

**Table 2:** Adverse events related to TACE from the second TACE procedure in the two groups before and after PSM analysis.

**Adverse events**	**Before PSM**		***P* value**	**After PSM**		***P* value**
	**TACE–apatinib (*n* = 126)**	**TACE-alone (*n* = 97)**		**TACE–apatinib (*n* = 90)**	**TACE-alone (*n* = 90)**	
Hepatorenal syndrome	2 (2%)	3 (3%)	0.767	1 (1%)	3 (3%)	0.613
Inguinal hematoma	3 (2%)	2 (2%)	>0.99	2 (2%)	2 (2%)	>0.99
Hepatic arterial dissection	1 (1%)	2 (2%)	0.819	1 (1%)	2 (2%)	>0.99
Pulmonary oil embolization	0 (0%)	1 (1%)	0.435	0 (0%)	1 (1%)	>0.99

*Data are numbers of events. Data in parentheses are percentages*.

*TACE, transarterial chemoembolization; PSM, propensity score matching*.

**Table 3:** Liver function changes at 4 weeks after treatment in the TACE–apatinib group before and after PSM analysis.

**Liver function**	**Before PSM**		***P* value**	**After PSM**		***P* value**
	**Pretreatment**	**At 4 weeks after treatment**		**Pretreatment**	**At 4 weeks after treatment**	
Total bilirubin level (μmol/L)	19.0 ± 7.8	19.2 ± 9.3	0.889	19.4 ± 9.4	19.1 ± 8.3	0.901
Serum albumin level (g/L)	36.5 ± 5.1	36.4 ± 5.4	0.915	37.1 ± 5.1	37.3 ± 5.0	0.732
Prothrombin time (s)	13.9 ± 1.0	13.9 ± 1.2	0.615	13.8 ± 1.0	13.9 ± 1.3	0.756

*Unless indicated, data are mean ± standard deviation*.

*TACE, transarterial chemoembolization; PSM, propensity score matching*.

The apatinib-related adverse events in the TACE–apatinib group are shown in Table 4. There were 283 adverse events occurring in 117 (93%) of the 126 patients. Fourteen grade 3 adverse events occurred in 14 patients, and all the 14 patients received an apatinib dose reduction or temporary interruption of drug administration. No grade 4 and 5 adverse events were observed. The symptoms related to the adverse events in these patients were relieved or eliminated after symptomatic treatments, drug reduction, or temporary interruption of drug administration. The administration of apatinib was continued until a patient had HCC in BCLC stage D, a patient's death, or a patient's withdrawal of consent from this study.

**Table 4:** Adverse events related to apatinib in the TACE–apatinib group before PSM analysis.

**Adverse events**	**Grade 1**	**Grade 2**	**Grade 3**	**Grade 4**	**Grade 5**	**All events**
Hand-foot skin reactions	30 (24%)	59 (47%)	8 (6%)	0 (0%)	0 (0%)	97 (77%)
Hypertension	40 (32%)	17 (13%)	1 (1%)	0 (0%)	0 (0%)	58 (46%)
Diarrhea	23 (19%)	6 (5%)	1 (0%)	0 (0%)	0 (0%)	30 (24%)
Fatigue	20 (16%)	8 (6%)	0 (0%)	0 (0%)	0 (0%)	28 (22%)
Headache	10 (8%)	5 (4%)	0 (0%)	0 (0%)	0 (0%)	15 (12%)
Oral ulcer	4 (3%)	5 (4%)	0 (0%)	0 (0%)	0 (0%)	9 (7%)
Voice change	6 (5%)	5 (4%)	0 (0%)	0 (0%)	0 (0%)	11 (9%)
Proteinuria	9 (7%)	20 (16%)	1 (1%)	0 (0%)	0 (0%)	30 (24%)
Gastrointestinal hemorrhage	1 (1%)	1 (1%)	2 (2%)	0 (0%)	0 (0%)	4 (4%)
New hypothyroidism	0 (0%)	0 (0%)	1 (1%)	0 (0%)	0 (0%)	1 (1%)

*Data are numbers of events. Data in parentheses are percentages*.

*TACE, transarterial chemoembolization; PSM, propensity score matching*.

### Efficacy Comparison Between the Groups of TACE–Apatinib and TACE Alone

**Figure 2:**
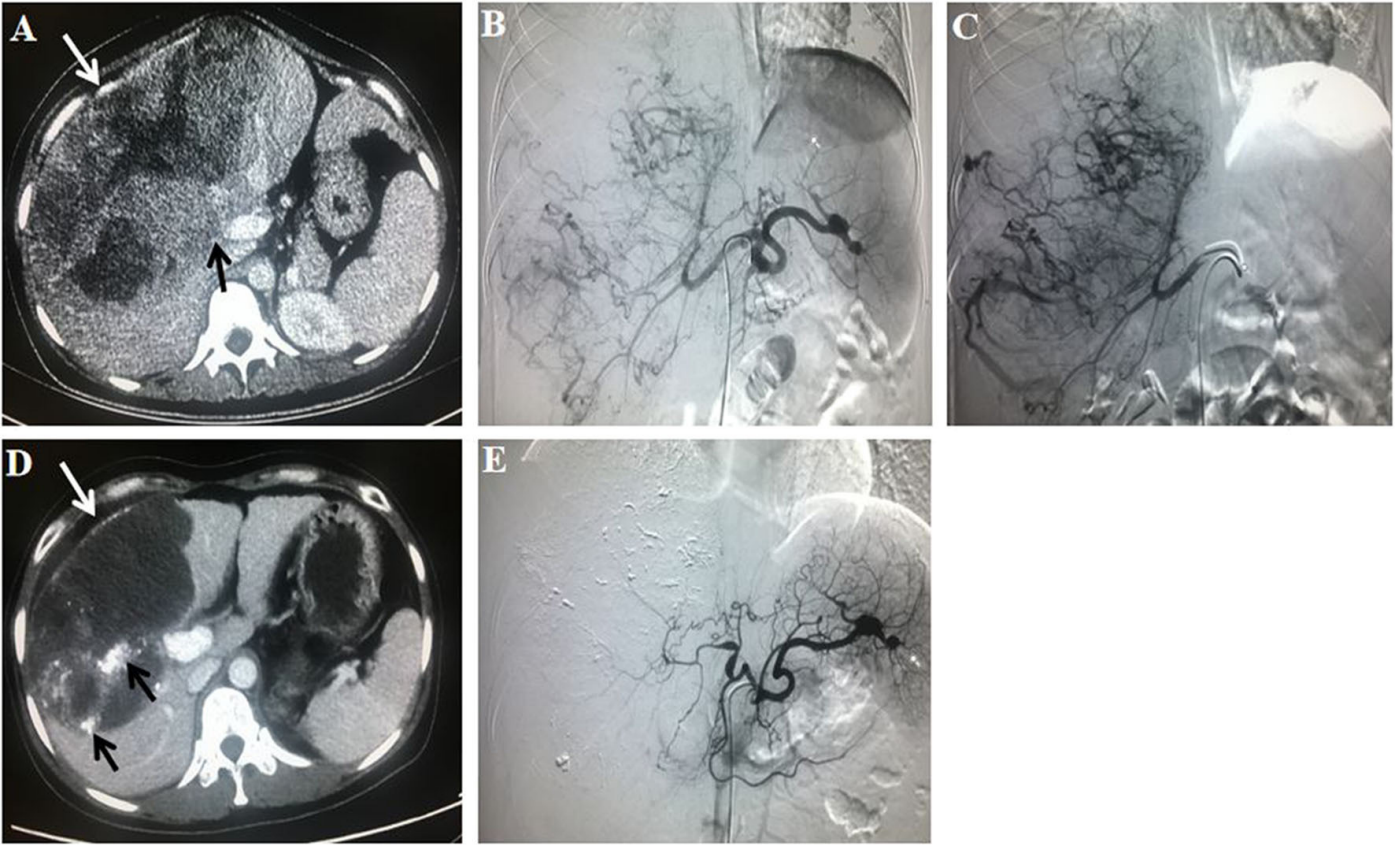
A 31-year-old female patient with Barcelona Clinic Liver Cancer stage C hepatocellular carcinoma who had a history of hepatitis B virus received transarterial chemoembolization (TACE)–apatinib treatment. The initial value of a-fetoprotein (AFP) was 570 ug/L. **(A)** A contrast-enhanced CT scan before the first TACE procedure showed a massive liver tumor (white arrow; size of 20.6 × 12.9 cm) which invaded the right branch of the portal vein (black arrow). **(B)** An angiography in the celiac trunk artery showed a mass of tumor-feeding arteries. **(C)** An angiography in the right hepatic artery showed that the tumor vessels were dilated and tortuous. **(D)** At 3 months after TACE–apatinib treatment, a contrast-enhanced CT scan showed that the tumor (white arrow), with lipiodol accumulation (black arrow), was significantly shrank (15.0 × 9.5 cm), and there was no enhancement within the tumor. **(E)** At 3 months after TACE–apatinib treatment, an angiography confirmed that there was no tumor in the liver. Tumor response according to the modified response evaluation criteria in solid tumors was complete response. Meanwhile, the value of AFP was decreased to normal (10.3 ug/L).

The tumor responses in patients with advanced HCC in the TACE–apatinib group and TACE-alone group are shown in Table 5. Before the PSM analysis, the DCR of tumor response in the TACE–apatinib group was significantly higher than that in the TACE-alone group (62 vs. 33%, *P* < 0.001). After the PSM analysis, the DCR of tumor response was 59% in the TACE–apatinib group, which was significantly higher than 33% in the group of TACE alone (*P* = 0.001). One representative case of TACE–apatinib in the treatment of BCLC stage C HCC is shown in Figure 2.

**Table 5:** Tumor responses at 3 months and AFP responses at 4 weeks after the first TACE treatment between the two groups before and after PSM analysis.

	**Tumor response**						**AFP response**					
	**Before PSM**		***P* value**	**After PSM**		***P* value**	**Before PSM**		***P* value**	**After PSM**		***P* Value**
	**TACE–apatinib**	**TACE alone**		**TACE–apatinib**	**TACE alone**		**TACE–apatinib**	**TACE alone**		**TACE–apatinib**	**TACE alone**	
	**(*n* = 126)**	**(*n* = 97)**		**(*n* = 90)**	**(*n* = 90)**		**(*n* = 62)**	**(*n* = 46)**		**(*n* = 47)**	**(*n* = 42)**	
Complete response (*n*)	5 (4%)	0 (0%)		4 (4%)	0 (0%)		2 (3%)	0 (0%)		2 (4%)	0 (0%)	
Partial response (*n*)	57 (45%)	10 (10%)		42 (47%)	9 (10%)		31 (50%)	8 (17%)		24 (51%)	8 (19%)	
Stable disease (*n*)	16 (13%)	22 (23%)		7 (8%)	21 (23%)		11 (18%)	10 (22%)		5 (11%)	9 (21%)	
Progressive disease (*n*)	48 (38%)	65 (67%)		37 (41%)	60 (67%)		18 (29%)	28 (61%)		16 (34%)	25 (60%)	
Disease control rate (%)	62%	33%	<0.001	59%	33%	<0.001	71%	39%	*P* = 0.001	66%	40%	*P* = 0.016

*Data in parentheses are percentages*.

*PSM, propensity score matching; TACE, transarterial chemoembolization; AFP, a-fetoprotein; n, number of patients*.

The AFP response at 4 weeks after the first TACE procedure was investigated for patients with an initial level of AFP greater than 400 ng/ml in the two groups (Table 5). Before the PSM analysis, the DCR of AFP response in the TACE–apatinib group was significantly higher than that in the TACE-alone group (71 vs. 39%, *P* = 0.001). After the PSM analysis, it was 66% in the group of TACE–apatinib, which was significantly higher than 40% in the group of TACE alone (*P* = 0.016).

**Figure 3:**
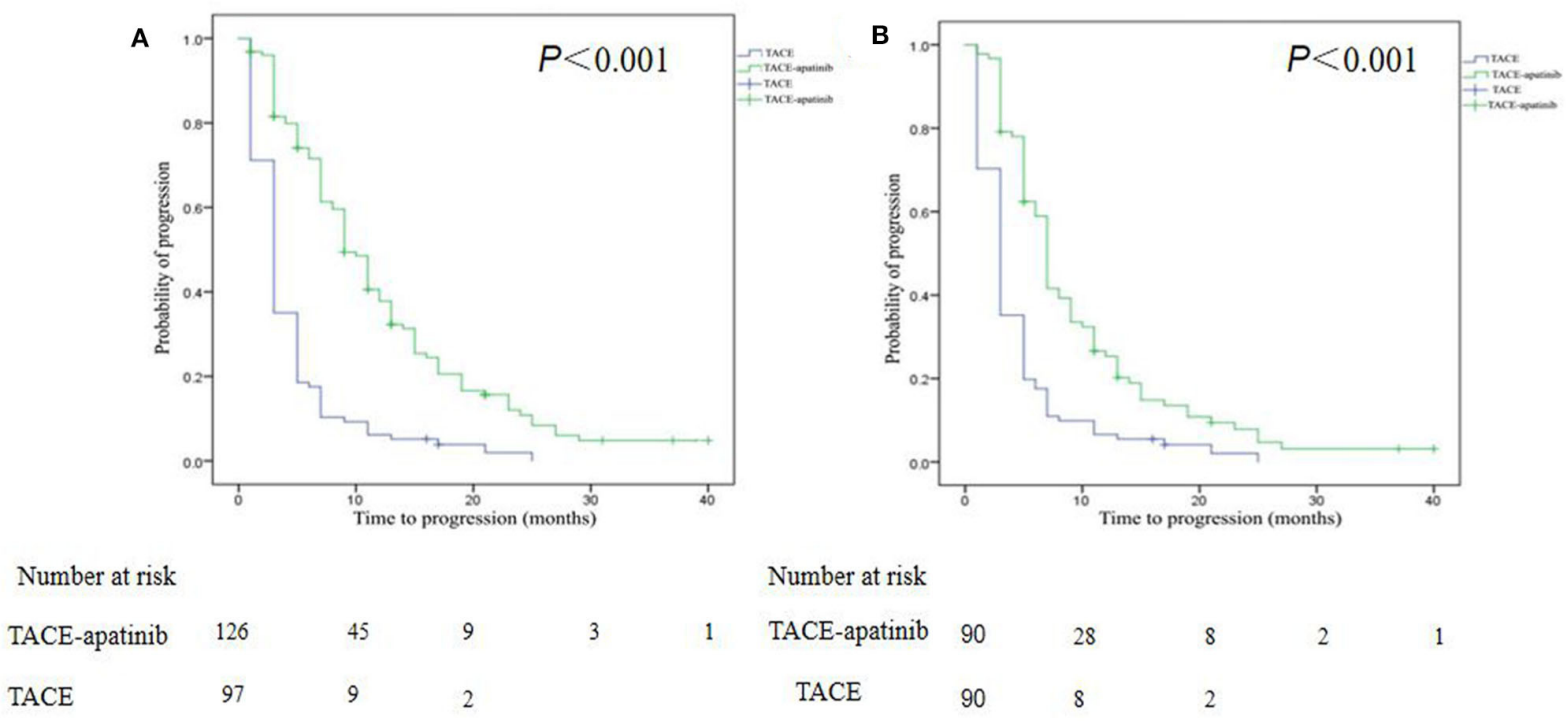
Kaplan–Meier curves of time to progression for patients with advanced hepatocellular carcinoma who received the treatment of transarterial chemoembolization (TACE)–apatinib or TACE alone before **(A)** and after **(B)** propensity score matching.

The median follow-up time was 9.0 months (range, 3.0–40.0 months) in the whole study. At the end of follow-up (March 31, 2019), 75% of patients in the TACE–apatinib group, and 97% of patients in the TACE-alone group died. Before the PSM analysis, the median TTP in the TACE–apatinib group was 9.0 months (95% CI, 7.6–10.4), and in the TACE-alone group it was 3.0 months (95% CI, 2.5–3.5). The median TTP between the two groups was significantly different (*P* < 0.001) (Figure 3A). After the PSM analysis, the median TTP in the TACE–apatinib group and the TACE-alone group were 7.0 months (95% CI, 6.6–7.4) and 3.0 months (95% CI, 2.5–3.5), respectively, and the difference between the two groups was significantly different (*P* < 0.001) (Figure 3B).

**Figure 4:**
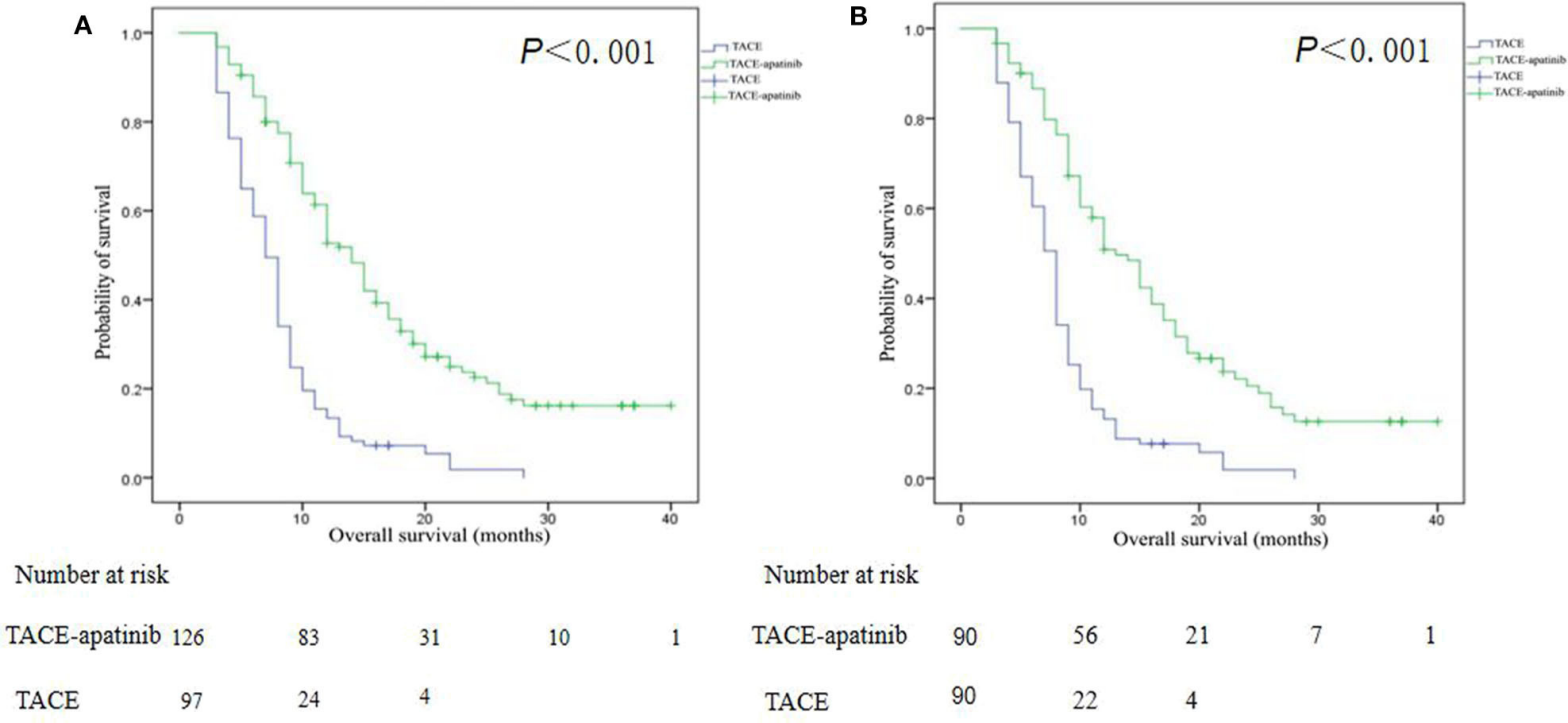
Kaplan–Meier curves of overall survival for patients with advanced hepatocellular carcinoma who received the treatment of transarterial chemoembolization (TACE)–apatinib or TACE alone before **(A)** and after **(B)** propensity score matching.

Before the PSM analysis, the median OS in the TACE–apatinib group was 14.0 months (95% CI, 12.1–15.9), and in the TACE-alone group it was 7.0 months (95% CI, 6.2–7.8). The median OS between the two groups was significantly different (*P* < 0.001) (Figure 4A). After the PSM analysis, the median OS in the TACE–apatinib group and the TACE-alone group were 13.0 months (95% CI, 10.3–15.7) and 8.0 months (95% CI, 7.3–8.7), respectively, and the difference between the two groups was significantly different (*P* < 0.001) (Figure 4B).

### Prognostic Factors Associated With OS

Univariate and multivariate analyses were performed to determine the prognostic factors for OS (Table 6). Before the PSM analysis, the univariate analysis indicated that Child–Pugh class A, absence of mild ascites, absence of PVTT, and the treatment method of TACE–apatinib were associated with better OS (*P* < 0.05). Moreover, the multivariate analysis indicated that the absence of mild ascites [hazards ratio (HR) = 0.68; 95% CI, 0.48–0.96; *P* = 0.029] and the treatment method of TACE–apatinib (HR = 0.34; 95% CI, 0.25–0.45, *P* < 0.001) were the independent protective factors for OS. After the PSM analysis, the univariate analysis revealed that the Child–Pugh class A and the treatment method of TACE–apatinib were significantly associated with better OS (*P* < 0.05). However, the multivariate analysis indicated that the treatment method of TACE–apatinib was the only independent protective factor for OS (HR = 0.35; 95% CI, 0.26–0.49, *P* < 0.001).

**Table 6:** Univariate and multivariate analysis of prognostic factors for overall survival (OS) before and after PSM analysis.

	**Univariate analysis**				**Multivariate analysis**			
	**Before PSM**		**After PSM**		**Before PSM**		**After PSM**	
**Variables**	**Median OS (95% CI)**	***P* value**	**Median OS (95% CI)**	***P* value**	**Hazards ratio (95% CI)**	***P* value**	**Hazards ratio (95% CI)r5**	***P* value**
Gender		0.729		0.396				
Male	10.0 (9.0, 11.0)		9.0 (8.0, 10.0)					
Female	7.0 (2.7, 11.3)		8.0 (2.9, 13.1)					
Age		0.074		0.267				
≤60	9.0 (6.8, 11.2)		9.0 (7.8, 10.2)					
>60	10.0 (8.5, 11.5)		9.0 (7.6, 10.4)					
ECOG performance		0.922		0.738			
1	9.0 (7.7, 10.3)		9.0 (7.8, 10.2)					
2	10.0 (7.2, 12.8)		10.0 (7.6, 12.4)					
HBV infection		0.402		0.972				
Yes	10.0 (8.8, 11.2)		9.0 (7.8, 10.2)					
No	10.0 (6.3, 13.7)		10.0 (6.6, 13.4)					
Child–Pugh class		0.046		0.034				
A	10.0 (8.7, 11.3)		9.0 (7.9, 10.1)					
B	5.0 (2.3, 7.7)		5.0 (3.0, 7.0)					
Mild ascites		0.001		0.111				
Absent	10.0 (8.5, 11.5)		9.0 (7.6, 10.4)					
Present	6.0 (3.1, 8.9)		8.0 (5.4, 10.6)					
AFP level (ng/ml)		0.184		0.678				
>400	10.0 (8.4, 11.6)		9.0 (7.7, 10.3)					
≤400	9.0 (7.3, 10.7)		9.0 (7.5, 10.5)					
Total bilirubin (μmol/L)		0.383		0.276				
≥34	9.0 (6.4, 11.6)		8.0 (5.3, 10.7)					
<34	10.0 (8.6, 11.4)		9.0 (7.7, 10.3)					
Albumin level (g/L)		0.492		0.693				
>35	10.0 (8.1, 11.9)		9.0 (7.4, 10.6)					
≤35	9.0 (7.3, 10.7)		9.0 (7.1, 10.9)					
PVTT		0.022		0.263				
Absent	11.0 (7.8, 14.2)		9.0 (6.4, 11.6)					
Present	9.0 (7.6, 10.4)		9.0 (7.6, 10.4)					
HVTT		0.584		0.578				
Absent	10.0 (9.0, 11.0)		9.0 (7.9, 10.1)					
Present	9.0 (7.2, 10.8)		9.0 (6.9, 11.1)					
Extrahepatic spread		0.119		0.159				
Absent	9.0 (7.6, 10.4)		8.0 (6.8, 9.2)					
Present	10.0 (8.0, 12.0)		10.0 (8.1, 11.9)					
Treatment method		<0.001		<0.001				
TACE–apatinib	14.0 (12.1, 15.9)		13.0 (10.3, 15.7)					
TACE	7.0 (6.2, 7.8)		8.0 (7.3, 8.7)					
Treatment method:								
TACE–apatinib					0.34 (0.25, 0.45)	<0.001	0.35 (0.26, 0.49)	<0.001
Child–Pugh class: A					0.68 (0.46, 1.0)	0.059	0.65 (0.42, 0.99)	0.058
Mild ascites: absent					0.68 (0.48, 0.96)	0.029	NA	-
PVTT: absent					0.77 (0.57, 1.04)	0.089	NA	-

*PSM, propensity score matching; ECOG, Eastern Cooperative Oncology Group; AFP, a-fetoprotein; TACE, transarterial chemoembolization; PVTT, portal vein tumor thrombus; HVTT, hepatic vein tumor thrombus*.

*-,no data; NA, not applicable*.

## Discussion

TACE is one of the most common treatment methods for unresectable HCC. However, incompletely destroying the tumors and tumor recurrence and metastasis after TACE, which resulted from the release of angiogenic cytokines from tumor cells after embolization (23), are the major disadvantages of TACE in the treatment of HCC. Sorafenib inhibited tumor growth, mainly relying on its effect of being anti-angiogenic (24). Therefore, some previous studies (9–11, 25, 26) focused on the combined treatment of TACE with sorafenib to potentially improve the treatment efficacy of advanced HCC. However, some disadvantages of sorafenib, such as the relatively high price for Chinese patients, side effects, and modest efficacy, limited its application in China. At present, new molecular targeted drugs for HCC are relatively few. Hence, a more appropriate and effective molecular targeted drug for Chinese patients with advanced HCC is urgently needed.

Apatinib is a powerful inhibitor of VEGFR-2 (27, 28). Through binding to VEGFR-2, apatinib displays anti-angiogenic effects and exhibits anti-tumor activity (29). A recent retrospective control study (30) showed that TACE–apatinib significantly increased the median TTP and the OS for patients with BCLC stage C HCC compared with TACE alone (median TTP, 6.3 vs. 3.5 months, *P* = 0.002; median OS, 13.0 vs. 9.9 months, *P* = 0.041). Meanwhile, two retrospective studies (31, 32) also reported that TACE–apatinib yielded encouraging efficacy for patients with advanced HCC. These results demonstrated that apatinib may improve the efficacy of TACE through inhibiting VEGFR-2 in patients with advanced HCC. Compared with these previous retrospective studies, our study included a relatively larger number of patients and had a smaller patient selection bias by using the PSM analysis. Therefore, the evidence level of TACE–apatinib in patients with advanced HCC in our study may be higher than those in previous studies.

The results of our study showed that the treatment method of TACE–apatinib was associated with better efficacy compared with TACE alone in the treatment of patients with advanced HCC before and after the PSM analysis. A higher DCR of tumor and AFP response and a significantly longer median TTP and median OS were observed in the TACE–apatinib group before and after the PSM analysis. The results of the univariate analysis before and after the PSM analysis showed that the treatment method of TACE–apatinib was associated with a significantly longer OS. Meanwhile, the multivariate analysis after the PSM analysis further demonstrated that the treatment method of TACE–apatinib was an independently favorable factor for better OS. These results verified our hypotheses that apatinib may improve the efficacy of TACE in patients with BCLC stage C HCC.

Generally speaking, apatinib can be tolerated by most patients and its related adverse events were not unacceptable for patients with advanced HCC. The observed apatinib-related adverse events in our study were hand–foot skin reactions, hypertension, diarrhea, fatigue, oral ulcer, headache, proteinuria, voice change, gastrointestinal hemorrhage, and new hypothyroidism. These adverse events were predominantly graded 1 or 2, and the symptoms that related to these adverse events can be relieved or eliminated after dose reduction or temporary interruption of drug administration and symptomatic treatments. Meanwhile, we found that there were no significant differences in TACE-related adverse events from the second TACE procedure between the two groups. This result indicated that apatinib did not increase the occurrence rate of TACE-related adverse events. Furthermore, we observed the changes of three representative indicators of liver function, and the values of total bilirubin, serum albumin, and prothrombin time at 4 weeks after the first TACE–apatinib did not change significantly compared with the baseline values at pre-treatment. This result indicated that apatinib did not significantly impact liver function when it was combined with TACE treatment. Therefore, these results of our study demonstrated that the treatment method of TACE–apatinib for patients with advanced HCC was safe.

Our study had limitations. First, our study was retrospective, and the sample size in the two treatment groups was relatively small. Although a PSM analysis was performed, the potential patient selection bias could not be completely avoided. Second, the data of our study came from a single center. An adequately powered multi-center prospective randomized trial of TACE–apatinib in patients with advanced HCC is necessary to confirm our findings.

## Conclusion

The efficacy of TACE–apatinib in patients with advanced HCC was inspiring, and the adverse events of apatinib were not unacceptable and uncontrollable for these patients.

## Data Availability Statement

The datasets used in this study are available from the corresponding author upon reasonable request.

## Ethics Statement

The studies involving human participants were reviewed and approved by The Ethics Committee of Tongji Medical College, Huazhong University of Science and Technology, Wuhan, China. The patients/participants provided their written informed consent to participate in this study.

## Author Contributions

FY and CZ contributed to the conception and the design of the study. XK, BL, FY, and CZ contributed to data acquisition, data analysis, and interpretation. XK and BL drafted and prepared the manuscript. All the authors made critical revisions of the draft versions of the manuscript and approved the final manuscript. All authors contributed to the article and approved the submitted version.

## Conflict of Interest

The authors declare that the research was conducted in the absence of any commercial or financial relationships that could be construed as a potential conflict of interest.
